# Exploring the Neandertal legacy of pancreatic ductal adenocarcinoma risk in Eurasians

**DOI:** 10.1186/s40659-023-00457-y

**Published:** 2023-08-13

**Authors:** Margherita Piccardi, Manuel Gentiluomo, Stefania Bertoncini, Raffaele Pezzilli, Bálint Erőss, Stefania Bunduc, Faik G. Uzunoglu, Renata Talar-Wojnarowska, Tomas Vanagas, Cosimo Sperti, Martin Oliverius, Mateus Nóbrega Aoki, Stefano Ermini, Tamás Hussein, Ugo Boggi, Krzysztof Jamroziak, Evaristo Maiello, Luca Morelli, Ludmila Vodickova, Gregorio Di Franco, Stefano Landi, Andrea Szentesi, Martin Lovecek, Marta Puzzono, Francesca Tavano, Hanneke W. M. van Laarhoven, Alessandro Zerbi, Beatrice Mohelnikova-Duchonova, Hannah Stocker, Eithne Costello, Gabriele Capurso, Laura Ginocchi, Rita T. Lawlor, Giuseppe Vanella, Francesca Bazzocchi, Jakob R. Izbicki, Anna Latiano, Bas Bueno-de-Mesquita, Ruggero Ponz de Leon Pisani, Ben Schöttker, Pavel Soucek, Péter Hegyi, Maria Gazouli, Thilo Hackert, Juozas Kupcinskas, Lina Poskiene, Matteo Tacelli, Susanne Roth, Silvia Carrara, Francesco Perri, Viktor Hlavac, George E. Theodoropoulos, Olivier R. Busch, Andrea Mambrini, Casper H. J. van Eijck, Paolo Arcidiacono, Aldo Scarpa, Claudio Pasquali, Daniela Basso, Maurizio Lucchesi, Anna Caterina Milanetto, John P. Neoptolemos, Giulia Martina Cavestro, Dainius Janciauskas, Xuechen Chen, Roger Chammas, Mara Goetz, Hermann Brenner, Livia Archibugi, Michael Dannemann, Federico Canzian, Sergio Tofanelli, Daniele Campa

**Affiliations:** 1 Department of Biology, Unit of Genetics, University of Pisa, Via Derna 1, 56126 Pisa, Italy; 2 Department of Biology, Unit of Zoology and Anthropology, University of Pisa, Pisa, Italy; 3 Potenza County Medical Association, Potenza, Italy; 4 Institute for Translational Medicine, Medical School, University of Pécs, Pécs, Hungary; 5 Center for Translational Medicine, Semmelweis University, Budapest, Hungary; 6 Division of Pancreatic Diseases, Heart and Vascular Center, Semmelweis University, Budapest, Hungary; 7 Carol Davila University of Medicine and Pharmacy, Bucharest, Romania; 8 Department of General, Visceral and Thoracic Surgery, University Medical Center Hamburg-Eppendorf, Hamburg, Germany; 9 Department of Digestive Tract Diseases, Medical University of Lodz, Lodz, Poland; 10 Department of Surgery, Lithuanian University of Health Sciences, Kaunas, Lithuania; 11 Department DISCOG, Chirurgia Generale 1, University of Padova, Padua, Italy; 12 Department of Surgery, Third Faculty of Medicine, University Hospital Kralovske Vinohrady, Charles University, Prague, Czech Republic; 13 Laboratory for Applied Science and Technology in Health, Carlos Chagas Institute, Curitiba, Brazil; 14 Blood Transfusion Service, Azienda Ospedaliero-Universitaria Meyer, Children’s Hospital, Florence, Italy; 15 Division of General and Transplantation Surgery, University of Pisa, Pisa, Italy; 16 Department of Hematology, Transplantation and Internal Medicine, University of Warsaw, Warsaw, Poland; 17 Department of Oncology, Fondazione IRCCS “Casa Sollievo Della Sofferenza” Hospital, San Giovanni Rotondo, Foggia Italy; 18 Department of Translational Research and New Technologies in Medicine and Surgery, General Surgery Unit, University of Pisa, Pisa, Italy; 19 Department of Molecular Biology of Cancer, Institute of Experimental Medicine of the Czech Academy of Sciences, Prague, Czech Republic; 20 Biomedical Center, Faculty of Medicine in Pilsen, Charles University, Pilsen, Czech Republic; 21 Institute of Biology and Medical Genetics, First Faculty of Medicine, Charles University, Prague, Czech Republic; 22 János Szentágothai Research Center, University of Pécs, Pécs, Hungary; 23 Department of Surgery I, University Hospital Olomouc, Olomouc, Czech Republic; 24 Gastroenterology and Gastrointestinal Endoscopy Unit, Vita-Salute San Raffaele University, IRCCS San Raffaele Scientific Institute, Milan, Italy; 25 Division of Gastroenterology and Research Laboratory, Fondazione IRCCS “Casa Sollievo Della Sofferenza” Hospital, San Giovanni Rotondo, Foggia, Italy; 26 Department of Medical Oncology, Amsterdam UMC, Cancer Center Amsterdam, University of Amsterdam, Amsterdam, The Netherlands; 27 Pancreatic Unit, IRCCS Humanitas Research Hospital, Milan, Italy; 28 Department of Biomedical Sciences, Humanitas University, Milan, Italy; 29 Department of Oncology, Faculty of Medicine and Dentistry, Palacky University, Olomouc, Czech Republic; 30 Division of Clinical Epidemiology and Aging Research, German Cancer Research Center (DKFZ), Heidelberg, Germany; 31 Network Aging Research (NAR), Heidelberg University, Heidelberg, Germany; 32 Department of Molecular & Clinical Cancer Medicine, University of Liverpool, Liverpool, UK; 33 Digestive and Liver Disease Unit, S Andrea Hospital, Rome, Italy; 34 Pancreas Translational and Clinical Research Center, Pancreato-Biliary Endoscopy and Endoscopic Ultrasound, San Raffaele Scientific Institute IRCCS, Milan, Italy; 35 Oncological Department, Oncology of Massa Carrara, ASL Toscana Nord Ovest, Massa Carrara, Italy; 36 ARC-NET Research Centre and Department of Diagnostics and Public Health, Section of Pathology, University of Verona, Verona, Italy; 37 Department of Surgery, Fondazione IRCCS “Casa Sollievo Della Sofferenza” Hospital, San Giovanni Rotondo, Foggia Italy; 38 Centre for Nutrition, Prevention and Health Services, National Institute for Public Health and the Environment (RIVM), Bilthoven, The Netherlands; 39 Pancreato-Biliary Endoscopy and Endoscopic Ultrasound, Pancreas Translational and Clinical Research Center, San Raffaele Scientific Institute IRCCS, Milan, Italy; 40 Laboratory of Biology, Medical School, National and Kapodistrian University of Athens, Athens, Greece; 41 Department of General, Visceral, and Transplantation Surgery, University of Heidelberg, Heidelberg, Germany; 42 Department of Gastroenterology and Institute for Digestive Research, Lithuanian University of Health Sciences, Kaunas, Lithuania; 43 Department of Pathology, Lithuanian University of Health Sciences, Kaunas, Lithuania; 44 Department of Gastroenterology, Endoscopic Unit, IRCCS Humanitas Research Hospital, Milan, Italy; 45 First Department of Propaedeutic Surgery, Hippocration General Hospital, National and Kapodistrian University of Athens, Athens, Greece; 46 Department of Surgery, Amsterdam UMC, Cancer Center Amsterdam, University of Amsterdam, Amsterdam, the Netherlands; 47 Department of Surgery, Erasmus MC University Medical Center, Rotterdam, the Netherlands; 48 Department DISCOG, Chirurgia Generale 3, University of Padova, Padua, Italy; 49 Department DIMED, Laboratory Medicine, University of Padova, Padua, Italy; 50 Medical Faculty Heidelberg, Heidelberg University, Heidelberg, Germany; 51 Department of Radiology and Oncology, Institute of Cancer of São Paulo (ICESP) São Paulo, Sao Paulo, Brazil; 52 Faculty of Medicine, University of São Paulo, São Paulo, Brazil; 53 Division of Preventive Oncology, German Cancer Research Center (DKFZ) and National Center for Tumor Diseases (NCT), Heidelberg, Germany; 54 German Cancer Consortium (DKTK), German Cancer Research Center (DKFZ), Heidelberg, Germany; 55 Estonian Biocentre, Institute of Genomics, University of Tartu, Tartu, Estonia; 56 Genomic Epidemiology Group, German Cancer Research Center (DKFZ), Heidelberg, Germany

**Keywords:** Neandertal, Pancreatic cancer, Association study, Introgression, Eurasians, Admixture

## Abstract

**Background:**

The genomes of present-day non-Africans are composed of 1–3% of Neandertal-derived DNA as a consequence of admixture events between Neandertals and anatomically modern humans about 50–60 thousand years ago. Neandertal-introgressed single nucleotide polymorphisms (aSNPs) have been associated with modern human disease-related traits, which are risk factors for pancreatic ductal adenocarcinoma (PDAC), such as obesity, type 2 diabetes, and inflammation. In this study, we aimed at investigating the role of aSNPs in PDAC in three Eurasian populations.

**Results:**

The high-coverage Vindija Neandertal genome was used to select aSNPs in non-African populations from 1000 Genomes project phase 3 data. Then, the association between aSNPs and PDAC risk was tested independently in Europeans and East Asians, using existing GWAS data on more than 200 000 individuals. We did not find any significant associations between aSNPs and PDAC in samples of European descent, whereas, in East Asians, we observed that the Chr10p12.1-rs117585753-T allele (MAF = 10%) increased the risk to develop PDAC (OR = 1.35, 95%CI 1.19–1.54, P = 3.59 × 10^–6^), with a P-value close to a threshold that takes into account multiple testing.

**Conclusions:**

Our results show only a minimal contribution of Neandertal SNPs to PDAC risk.

**Supplementary Information:**

The online version contains supplementary material available at 10.1186/s40659-023-00457-y.

## Introduction

By comparing the genome sequences of Neandertal and modern genomes it has been shown that ~ 1–3% of the genomes of present-day non-Africans are of Neandertal ancestry [1–3] with 8–20% higher levels of Neandertal ancestry in East Asians compared to Europeans [4–6]. Through phenotypic information from genome-wide association studies it has been shown that introgressed Neandertal DNA still significantly influences the phenotypic variability of anatomically modern humans (AMHs) today. Neandertal-introgressed Single Nucleotide Polymorphisms (aSNPs) have for example been associated with several human traits, such as the genetic susceptibility of type 2 diabetes (T2D), obesity, age of menopause, neurological traits, morning preference, skin and hair morphology, immune response, and inflammation [7–17]. Among these traits are several factors, such as overweight, obesity, T2D, deregulation of the immune system, and chronic inflammation that play a key role in pancreatic ductal adenocarcinoma (PDAC) onset and progression [18–21].

Alongside a small number of environmental risk factors [22–24], PDAC susceptibility has a strong genetic component. Rare high penetrance variants involved in hereditary syndromes (reviewed in Gentiluomo et al.) and frequent low and moderate penetrance variants, discovered through candidate gene and genome-wide association studies (GWAS), have been identified as playing a role in PDAC onset [25–39]. However, the common risk loci discovered so far explain only a small proportion of the overall heritability of the disease [40]. Furthermore, PDAC is a late onset disease [29, 41, 42], thus loci associated with PDAC susceptibility tend to persist in the AMH gene pool, eluding purifying selection.

Considering that aSNPs are associated with several PDAC risk factors and that the genetic contribution to PDAC etiology still needs to be elucidated, we aimed at investigating the Neandertal legacy of PDAC genetic risk. We analysed PDAC GWAS cohorts from different Eurasian populations for significant associations with aSNPs to study the role of Neandertal admixture and PDAC risk in different ancestry groups. This study is the first attempt to investigate the role of archaic admixture on PDAC development.

## Results

In this study, 389 144 aSNPs were identified among the non-African populations of the 1000 Genomes project [43]. The association between aSNPs and the risk of developing PDAC was tested in three ancestry groups: non-Finnish Europeans, Finns, and East Asians.

**Fig. 1:**
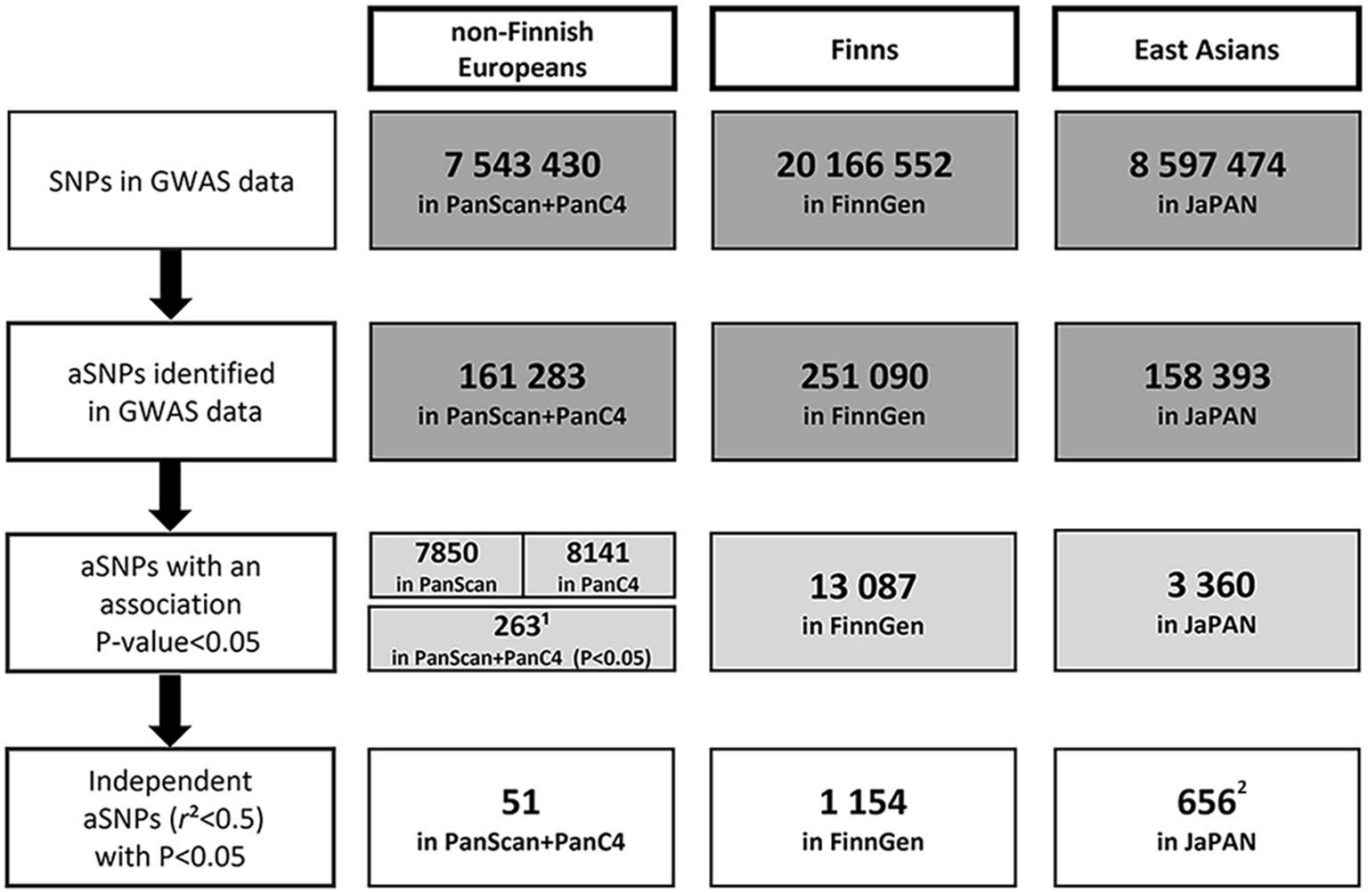
aSNPs filtering and analysis workflow for each ancestry group. The figure displays aSNPs analysis workflow for non-Finnish Europeans, Finns, and East Asians. The 389 144 aSNPs identified in all non-African populations from 1000 Genomes project phase 3, were filtered and analysed for each ancestry group. *aSNP* Neandertal introgressed SNP. ^1^aSNPs that showed an association P-value < 0.05 in PanScan, PanC4 and in the two datasets combined. aSNPs with a P < 0.05 in PanScan (7850), PanC4 (8141), and the combined datasets (8718). ^2^aSNPs with a P-value < 0.05 and an identical direction of the effect in all the three GWASs included in JaPAN

For non-Finnish Europeans, 161 283 aSNPs were available to be analysed in the discovery phase, using the genotypes of PanScan + PanC4 studies. Considering a P < 0.05, 263 aSNPs resulted associated with PDAC risk in the combined PanScan + PanC4 dataset. All 263 of these aSNPs also passed the P < 0.05 threshold when only PanScan or PanC4 were considered separately. Among them, 212 showed residual LD (r^2^ > 0.5). After pruning, 51 independent aSNPs associations spanning across 51 loci were observed (Fig. 1, Additional File 1). None of the 51 aSNPs remained associated with PDAC after correction for multiple testing (p_j_ = 2.30 × 10^–6^). The SNP with the lowest P-value was Chr2p14-rs12998719, (OR = 1.11, 95%CI 1.05–1.16, P = 5.51 × 10^–5^) (Table 1). This variant has been already reported to be associated with PDAC risk [32] and was genotyped in the context of the PANDoRA consortium (replication phase). The results of the replication phase did not show a statistically significant association (OR = 1.46, 95%CI 0.95–1.15, P = 0.38) (Table 1).

**Table 1:** Candidate aSNPs for each ancestry group

Panel A. Association studies results								
Population	aSNP	Position (GRCh37)	Alleles (M/m)	Gene^a^	MAF^b^	OR (95%C.I.)	P	
PanScan + PanC4^c^	rs12998719	2:67583252	G/A	–	29%	1.11 (1.05–1.16)	5.51 × 10^–5^	
PANDoRA^c^	rs12998719	2:67583252	G/A	–	28%	1.05 (0.95–1.15)	0.38	
FinnGen	rs113955626	3:23107748	C/T	–	7%	1.35 (1.17–1.55)	4.79 × 10^–5^	
JaPAN	rs117585753	10:25190572	C/T	*PRTFDC1*	10%	1.35 (1.19–1.54)	3.59 × 10^–6^	

Panel B. Top SNP population-based frequency											
Population	aSNP	Position (GRCh37)	Alleles (M/m)	1000G MAF			gnomAD MAF			HGDP^d^	
				EUR	FIN	EAS (%)	EUR (%)	FIN	EAS (%)	EUR (%)	EAS
PanScan + PanC4^c^	rs12998719	2:67583252	G/A	27%	30%	43	36	29%	42	25	35%
FinnGen	rs113955626	3:23107748	C/T	4%	4%	13	5	8%	14	8	18%
JaPAN	rs117585753	10:25190572	C/T	8‰	5‰	10	1	3‰	9	1	8%

Panel A shows the summary statistics of aSNPs with the lowest association P-value, panel B shows the minor allele frequency of SNPs with the lowest P-value of associations across several datasets

*aSNP* Neandertal introgressed SNP; *M* major allele; m, minor allele; *MAF* minor allele frequency; *OR* Odds Ratio; *95%C.I*. 95% Confidence Interval; *PRTFDC1* phosphoribosyl transferase domain containing 1; *1000G* 1000 Genomes project phase 3 [43]; *gnomAD* Genome Aggregation Database [44]; *HGDP* Human Genome Diversity Project [45] *EUR* Europeans; *FIN* Finns; *EAS* East Asians

^**a**^Gene in which aSNP lies

^b^MAF of the SNP of interest in the analysed datasets. Since allele frequencies are not freely available in JaPAN dataset, the MAF in East Asians from 1000G is reported

^c^PanScan + PanC4 (discovery phase), PANDoRA (replication phase)

^d^In HGDP, SNP frequency in Finns is not available. Frequency in all European populations is displayed

In FinnGen, 251 090 aSNPs were found, and after LD-pruning (r^2^ > 0.5), 1154 independent aSNPs with a P < 0.05 were observed (Fig. 1). The aSNP with the lowest P-value in FinnGen was Chr3p24.3-rs113955626 (OR = 1.35, 95%CI 1.17–1.55, P = 4.79 × 10^–5^) (Table 1); this aSNP did not reach the Bonferroni adjusted significance threshold (p_j_ = 2.30 × 10^–6^).

In the JaPAN dataset, which includes data of the meta-analysis of three GWAS conducted on individuals of Asian descent, 158 393 aSNPs were analysed. The association analysis showed 656 independent aSNPs with a P < 0.05 in all the three GWASs (Fig. 1). The best candidate was Chr10p12.1-rs117585753 (OR = 1.35, 95%CI 1.19–1.54, P = 3.59 × 10^–6^), whose P-value was very close to the Bonferroni-adjusted threshold (p_j_ = 2.28 × 10^–6^) (Table 1).

## Discussion

We tested the effects of Neandertal introgression on PDAC susceptibility in three ancestry groups. In non-Finnish Europeans and Finns, no novel significant associations between aSNPs and PDAC were observed.

In JaPAN, we found that the T allele of Chr10p12.1-rs117585753 increased the risk to develop PDAC (P = 3.59 × 10^–6^). This association was not statistically significant when considering multiple testing. However, it is very close to the Bonferroni corrected threshold (p_j_ = 2.28 × 10^–6^). The functional implications of this aSNP have not been clarified yet: according to GWAS catalog, it is not associated with any complex human trait.

Interestingly, the T allele of Chr10p12.1-rs117585753 is present in EAS (MAF = 10%), whereas it is almost absent in the other populations represented in 1000 Genomes project (e.g., MAF < 0.01 in Europeans from 1000 Genomes project). Since Chr10p12.1-rs117585753 is polymorphic only in Asians, it is possible that the role of this aSNP in complex traits has not been elucidated yet because most of the association studies have been conducted in cohorts with participants of European descent [46]. The lower number of studies with Asian individuals implies that the associations between SNPs, which are rare in Europeans but common in Asians, still need further investigation to be understood entirely.

Chr10p12.1-rs117585753 lies in an intron of the protein-coding *PRTFDC1* gene, in which, according to the GWAS catalog, there are SNPs associated with blood cell count [47–49]. Several white and red blood cell count parameters have been used to predict immune response and inflammation in various diseases, including PDAC [50, 51]. One SNP in *PRTFDC1* (Chr10p12.1-rs7905553) is in weak LD (*r*^*2*^ = 0.14, D’ = 0.96) with Chr10p12.1-rs117585753 in EAS, and according to GWAS catalog it is associated with red blood cell distribution width (RDW) [52], which is a parameter of erythrocyte variation. RDW has been proposed as a biomarker of the inflammatory state that could predict progression/prognosis in PDAC [53], suggesting a potential contribution of the *PRTFDC1* genomic region and Chr10p12.1-rs117585753 in PDAC and immunity.

Several Neandertal-derived haplotypes involved in immunity have been reported to be under selection after Neandertal-AMH introgression. In fact, the positive selection of aSNPs that lead to adaptation (adaptive introgression) has been observed to be driven by the immune response to pathogens [8, 9, 54–57].

Possible limitations of our approach are represented by the fact that we could have underestimated the role of rare variants (MAF < 1%) because we did not have enough statistical power to detect associations between rare aSNPs and PDAC, although we used the largest PDAC datasets currently available, which included more than 200 000 individuals of three different ancestries. An additional potential limitation of this work is that 93 695 out of 389 144 aSNPs identified in Eurasian genomes could not be found in PanScan + PanC4, FinnGen, and JaPAN. Therefore, the role of these aSNPs in PDAC susceptibility was not explored. In future analyses, larger reference panels for imputation could be used to maximize the investigated Neandertal-derived genetic variability.

## Conclusions

In conclusion, we observed that the Neandertal introgressed DNA does not influence PDAC susceptibility in populations of European descent. Interestingly, we observed a potential association between Chr10p12.1-rs117585753-T and an increased risk of developing PDAC in populations of Asian descent, although not formally significant after correction for multiple testing. This aSNP is polymorphic only in East Asians and is situated in a genomic region involved in immunity. Further investigations are needed to elucidate the evolutionary processes that lead to these aSNPs in the AMH gene pool and the role of aSNPs in PDAC risk, and more broadly, to explore the Neandertal legacy in the susceptibility to other cancer types.

## Methods

### Neandertal SNPs identification

The method to select aSNPs was previously described [12]. Briefly, to define a potential introgressed allele, we used four criteria that needed to be fulfilled: (a) the allele is shared between the Vindija Neandertal [5] and at least one non-African population from 1000 Genomes project phase 3 [43]; (b) the allele is not present in Yoruba from sub-Saharan Africa; (c) the allele is carried in homozygous state by Vindija Neandertal; (d) based on the haplotype length, the allele is more likely derived from Neandertal-AMH admixture than incomplete lineage sorting (ILS). To apply the fourth criterion, an approach, that was previously described by Huerta-Sánchez et al*.,* and Dannemann et al. was used [54, 58]. Briefly, it allows the identification of putative Neandertal introgressed regions in all non-African 1000 Genomes project populations. Two recombination maps [59, 60] were used to calculate the expected ILS segments length based on the local recombination rate. Then, the probability that a segment length was consistent with ILS was computed and the resulting P-values were corrected through Benjamini–Hochberg method. Haplotypes that showed an adjusted P-value < 0.05 were considered as introgressed from Neandertal. The aSNPs used in the following analyses lay on one of these Neandertal-derived haplotypes.

All the analyses were based on human genome assembly GRCh37, and only biallelic loci were considered, excluding indels.

### Study populations

The association between aSNPs and PDAC risk was tested in three ancestry groups: non-Finnish Europeans, Finns and East Asians. A two-phase association study (discovery and replication) was performed to examine if aSNPs identified in non-Finnish Europeans affected PDAC susceptibility. On the other hand, a validation set was not available for Finns and East Asians, and the association between aSNPs and PDAC was tested by searching for aSNPs in FinnGen and JaPAN datasets, respectively (see below).

For non-Finnish European analyses, the discovery set included data of the Pancreatic Cancer Cohort Consortium (PanScan) and the Pancreatic Cancer Case–Control Consortium (PanC4). The data were downloaded from the database of Genotypes and Phenotypes (dbGaP, https://www.ncbi.nlm.nih.gov/gap/). The dbGaP study accession numbers were: phs000206.v5.p3 and phs000648.v1.p1.; the project reference number was #12644. Details about data collection, genotyping methods and analyses are described in the original publications [26, 31, 32, 61].

Genotype data were imputed separately, for each dataset, using the Michigan Imputation Server (https://imputationserver.sph.umich.edu) [62] and the Haplotype Reference Consortium (HRC, V.r1.1) as reference panel [63]. Prior to the imputation, the following quality controls were applied: genotypes missingness (call rate < 0.9), heterozygosity (> 3 SD from the mean), relatedness (PI_HAT > 0.2), PCA outliers (using PCA), and Hardy–Weinberg equilibrium (P < 1 × 10^−6^). After imputation, SNPs with low imputation quality (INFO score r^2^ < 0.7) were excluded. Finally, the imputed datasets were merged. A total of 7 543 430 SNPs passed the quality controls on the autosomal genome, and 8738 PDAC cases and 7034 controls were used in the analysis (Table 2).

**Table 2:** Study population description for each ancestry group

Population	Cases	Controls	Sex		Median age (25–75% percentile)		Total number of subjects
			Male	Female	Cases	Controls	
*non-Finnish Europeans*							
PanScan + PanC4^a^	8738	7034	54%	46%	65 (55–75)	65 (55–75)	15 772
PANDoRA^a^	1894	3089	51%	49%	67 (59–73)	59 (49–67)	4983
*Finns*							
FinnGen	1249	259 583	–	–	–	–	260 832
*East Asians*							
JaPAN^b^	2039	32 592	49.3–62.6%	–	62.7–66.3	43.6–56.3	34 631

The table shows the number of cases and controls in PanScan + PanC4, PANDoRA, FinnGen and JaPAN. Male and female count and median age of cases and controls are displayed for each study

^a^PanScan + PanC4 (discovery phase), PANDoRA (replication phase)

^b^Data for male count and age are displayed as minimum–maximum values of the three GWASs included in JaPAN

Data not available: “- “

The replication of aSNPs with a P-value of association with PDAC risk lower than the Bonferroni-adjusted threshold (see below) was attempted in the Pancreatic Disease Research (PANDoRA) consortium [64, 65]. PANDoRA is a multicentric study on pancreatic cancer based mainly on European countries (Greece, Italy, Germany, Netherlands, Denmark, Czech Republic, Hungary, Poland, Ukraine, Lithuania, UK). In addition, PANDoRA includes a subgroup of Brazilian cases and controls that were excluded from the validation set in this study because PanScan + PanC4 (discovery set) included only Caucasian samples, while Brazilians belong to different ancestries (unlike the other PANDoRA samples). Information on sex, and age (recruitment for controls and diagnosis for the cases) was collected for each participant. The controls were enrolled among the general population, blood donors or hospitalised individuals not affected by cancer, chronic pancreatitis, or diabetes [64]. For this study, 4983 individuals (1894 PDAC cases and 3089 controls) from PANDoRA were included in the analysis (Table 2).

Non-Finnish Europeans and Finns were analysed separately because PanScan + PanC4 and PANDoRA mainly include subjects with Central European ancestry. We used the FinnGen Release 8 (R8) data that consists of GWAS summary statistics of 1249 pancreatic cancer cases and 259 583 controls with Finnish ancestry (Table 2). Subjects affected by other cancer types were excluded from the controls (https://FinnGen.gitbook.io/documentation/) [66].

To examine the association between aSNPs identified in East Asians and PDAC, we downloaded JaPAN consortium dataset that consisted of summary statistics of a meta-analysis of three GWASs (JaPAN, National Cancer Center and BioBank Japan GWASs). Comprehensive information on genotyping and data analysis are given in the original publication [67]. Summary statistics for the GWAS analysis are available on the JaPAN consortium website (http://www.aichi-med-u.ac.jp/JaPAN/current_initiatives-e.html) and include 34 631 individuals of East Asian origin (2039 PDAC cases and 32 592 controls) (Table 2).

### Data and statistical analyses

**Fig. 2:**
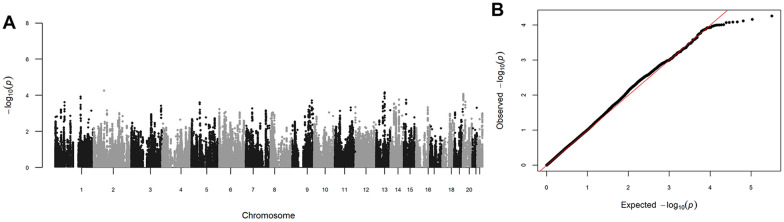
Manhattan and Quantile–Quantile (Q-Q) plots of PanScan + PanC4 association study results. The P-values displayed in Manhattan (**A**) and Q-Q plots (**B**) are calculated combining PanScan and PanC4 datasets. The plots were done using *qqman* R package (https://cran.r-project.org/web/packages/qqman/index.html) [68]. The inflation factors (l) did not indicate systematic inflation for PanScan (l = 1.02), PanC4 (l = 1.05), and combined datasets (l = 1.05). The inflation factors were computed using *simtrait* R package (https://cran.r-project.org/web/packages/simtrait/index.html) [69]

For non-Finnish Europeans, the association between aSNPs and PDAC susceptibility was tested in the PanScan + PanC4 dataset using logistic regression analysis, adjusting for age, sex and the top eight principal components (Fig. 2). To obtain a list of independent aSNPs, all aSNPs in linkage disequilibrium (LD; r^2^ > 0.5) with each other were excluded, and in each LD block the aSNP with the lowest association P-value was selected. Then, all aSNPs showing an association lower than the threshold for statistical significance corrected for multiple testing in PanScan, PanC4 and in the combined datasets were selected for replication in PANDoRA.

The genomic DNA of the PANDoRA samples was extracted from circulating blood using the QIamp^®^ 96 DNA QIcube^®^ HT Kit (Qiagen, Hilden, Germany). The genotyping was done using TaqMan RealTime PCR assays in 384-well plates. Each plate included cases and controls, duplicated samples for quality controls (QCs) and negative controls. The fluorescent signal detection was detected through a QuantStudioTM 5 Real-Time PCR system (Thermofisher, USA) and genotypes were called using the QuantStudio™ Design and Analysis Software v1.5.1. Samples with a genotyping call rate lower than 75% were excluded from the analysis. Hardy–Weinberg equilibrium test was performed with the Pearson chi-square test. To test the association between aSNPs and PDAC risk in PANDoRA, a logistic regression adjusted for age, sex, and country of origin was used.

For Finns and East Asians, the analyses were carried out in parallel, keeping separated the two ancestry groups. Considering that for FinnGen and JaPAN we used summary statistics, we looked at the P-value for association in these two datasets for the aSNPs selected for the two populations. Since JaPAN is a meta-analysis of three studies, along with P-value, the concordance of the direction of the effect between the three GWASs was considered.

P-value correction for multiple testing was performed using Bonferroni correction and considering the independent (*r*^*2*^ < 0.8) aSNPs. The adjusted significance thresholds were: 0.05/19 623 = 2.55 × 10^–6^ for PanScan + PanC4 and PANDoRA; 0.05/21 780 = 2.30 × 10^–6^ for FinnGen; 0.05/21 965 = 2.28 × 10^–6^ for JaPAN.

### Supplementary Information

**Additional file 1: **Title of data: Independent associations (P<0.05) identified in PanScan+PanC4 association studiesDescription of data: Independent (r^2^<0.5) aSNPs showing an association P<0.05 in PanScan, PanC4, and combined datasets. The displayed summary statistics and MAF are referred to the analyses with the combined datasets. (*Abbreviations: *aSNP, Neandertal introgressed Single Nucleotide Polymorphism; m, minor allele; M, major allele*, *MAF, minor allele frequency).

## Data Availability

The PanScan and PanC4 genotyping data are available from the dbGaP website (study accession numbers phs000206.v5.p3 and phs000648.v1.p1). The JaPAN data used in this work are available in the JaPAN consortium website (http://www.aichi-med-u.ac.jp/JaPAN/current_initiatives-e.html). FinnGen R8 dataset of PDAC cases and controls (“C3_PANCREAS_EXALLC”) is downloadable from the FinnGen official website (https://finngen.gitbook.io/documentation/). The PANDoRA data for this work will be made available to researchers who submit a reasonable request to the corresponding author, conditional to approval by the PANDoRA Steering Committee and Ethics Commission of the Medical Faculty of the University of Heidelberg. Data will be stripped from all information allowing identification of study participants.

## Abbreviations

95%C.I.: 95% Confidence interval
AMH: Anatomically modern human
aSNP: Neandertal intogressed single nucleotide polymorphism
dbGaP: Database of genotypes and phenotypes
EAS: East Asians from 1000 genomes project phase 3
EUR: Europeans from 1000 genomes project phase 3
gnomAD: Genome aggregation database
GWAS: Genome-wide association study
HGDP: Human genome diverity project
HRC: Haplotype Reference Consortium
ILS: Incomplete lineage sorting
JaPAN: Japan Pancreatic Cancer Research consortium
LD: Linkage disequilibrium
MAF: Minor allele frequency
OR: Odds ratio
PanC4: Pancreatic Cancer Case Control consortium
PANDoRA: Pancreatic Disease Research consortium
PanScan: Pancreatic Cancer Cohort consortium
PCA: Principal component analysis
PDAC: Pancreatic ductal adenocarcinoma
QC: Quality controls
Q-Q plot: Quantile–quantile plot
RDW: Red blood cell distribution width
T2D: Type 2 diabetes
