# Supplementary material for: Exploring the Neandertal legacy of pancreatic ductal adenocarcinoma risk in Eurasians
Source: Biol Res. 2023 Aug 13;56:46. doi: 10.1186/s40659-023-00457-y (PMC10424372; doi:10.1186/s40659-023-00457-y)
Supplement: Supplementary file 1 — Additional file 1: Title of data: Independent associations (P<0.05) identified in PanScan+PanC4 association studiesDescription of data: Independent (r2<0.5) aSNPs showing an association P<0.05 in PanScan, PanC4, and combined datasets. The displayed summary statistics and MAF are referred to the analyses with the combined datasets. (Abbreviations: aSNP, Neandertal introgressed Single Nucleotide Polymorphism; m, minor allele; M, major allele, MAF, minor allele frequency). [file 40659_2023_457_MOESM1_ESM.pdf]

Additional File 1. Independent associations (P<0.05) identified in PanScan+PanC4 association studies.
Independent (r^2>0.5) aSNPs showing an association P<0.05 in PanScan, PanC4, and combined datasets. The displayed summary statistics resulted from the analysis with the combined datasets. aSNPs are arranged in increasing order of P-value. (*Abbreviations:* aSNP, Neandertal introgressed Single Nucleotide Polymorphism; m, minor allele; M, major allele, MAF, minor allele frequency).

aSNP	position	M/m	OR (95%C.I.)	P	MAF	aSNPs in LD with SNP in first column
rs12998719	2:67583252	G/A	1.11 (1.05-1.16)	5.51E-05	0.29	-
rs61966285	13:73754491	A/G	1.2 (1.1-1.31)	6.92E-05	0.07	rs75374843; rs61966283; rs61966270; rs61966272; rs61966278; rs61966276; rs61966275; rs61966315: rs61967426; rs61967427; rs61967420
rs72637203	1:163826043	T/C	0.81 (0.72-0.9)	1.49E-04	0.04	-
rs11621061	14:73993369	C/T	0.9 (0.85-0.95)	1.75E-04	0.22	rs61987098; rs112943342; rs55746412; rs61987093; rs61987092; rs61988864; rs7159760; rs62004868; rs145538420; rs8008784; rs7158675; rs56175115; rs61988862; rs7157889; rs61988863; rs17182412
rs75150422	13:63320439	T/C	0.66 (0.53-0.82)	2.29E-04	0.01	rs78065501; rs148797436; rs76176688; rs149174916
rs117985418	20:17476761	G/A	0.73 (0.61-0.86)	2.30E-04	0.02	rs78925871; rs74488569; rs139487395
rs78759798	1:61844428	C/T	1.16 (1.07-1.26)	2.43E-04	0.09	rs77394732; rs17356741; rs114939720; rs17265629
rs78390364	5:53951839	G/A	0.8 (0.71-0.9)	2.53E-04	0.04	rs185489626; rs150512284; rs80170612
rs12897922	14:43001228	C/A	1.2 (1.09-1.33)	2.94E-04	0.05	rs12877994; rs12878899; rs12896511; rs66491209; rs35631271; rs35210813; rs12891764; rs35133792; rs35646082; rs12893585; rs34157262; rs34221614; rs67108906; rs72676360; rs17112972
rs75342815	15:39187846	A/C	1.32 (1.13-1.53)	2.98E-04	0.02	-
rs76264672	9:110909694	C/T	0.83 (0.75-0.92)	3.96E-04	0.05	rs117199844; rs116943216; rs10979225; rs78645898; rs12340710; rs77712704; rs78879621; rs12343590; rs75119583; rs12342378; rs77543845; rs12338325; rs79661666; rs77528613; rs116915917
rs77139395	3:191445560	C/T	1.17 (1.07-1.29)	5.24E-04	0.07	rs77199312; rs75047958
rs147979801	7:41387183	T/A	0.85 (0.78-0.93)	5.36E-04	0.06	rs116903293; rs78315069; rs11767732; rs55988998; rs117626946; rs77454616; rs17624981
rs113831293	6:1080941	T/C	0.85 (0.78-0.93)	5.84E-04	0.06	-
rs80218418	1:39191712	C/G	0.82 (0.73-0.92)	6.22E-04	0.04	rs149925270
rs74890892	9:103994550	A/G	0.77 (0.67-0.9)	6.48E-04	0.02	rs117857762; rs117938445; rs117523034; rs74988536; rs74570992; rs79025031; rs76677606

Continues in the next page

aSNP	position	M/m	OR (95%C.I.)	P	MAF	aSNPs in LD with SNP in first column
rs4649395	1:232763275	G/T	1.48 (1.18-1.85)	7.14E-04	0.01	-
rs55955026	7:129441957	T/A	0.87 (0.81-0.94)	7.67E-04	0.09	-
rs17759656	6:130405923	C/T	1.14 (1.05-1.23)	9.04E-04	0.10	rs13204752; rs34552886; rs62421345; rs62421346; rs13196495; rs17816084; rs17705246; rs1932103; rs10485401
rs17515149	8:16853661	C/T	1.15 (1.06-1.25)	9.11E-04	0.08	-
rs4804790	19:7797154	G/T	1.16 (1.06-1.26)	9.15E-04	0.07	rs117034642; rs4804798; rs76560987; rs117133715; rs4804799
rs13209147	6:33792499	G/A	0.91 (0.86-0.96)	9.51E-04	0.21	-
rs62168909	2:163761091	G/A	1.17 (1.07-1.28)	9.91E-04	0.06	rs62168910; rs1972672
rs2858635	22:49646062	T/C	0.89 (0.82-0.95)	1.01E-03	0.11	rs2688139; rs2858634; rs2688140; rs2688134; rs2858640; rs2688133; rs2858637; rs2858639; rs2688136; rs2688135; rs2001074
rs76078234	6:1370689	G/C	1.15 (1.06-1.24)	1.02E-03	0.09	rs77160910; rs75294181; rs78915578
rs78051589	8:9216948	C/G	1.13 (1.05-1.22)	1.15E-03	0.11	rs76155946; rs12155604
rs7264182	20:3691738	T/A	1.18 (1.07-1.3)	1.29E-03	0.06	-
rs140855472	14:94454139	A/T	1.25 (1.09-1.43)	1.35E-03	0.03	-
rs75720706	1:98006414	A/T	0.75 (0.63-0.9)	1.40E-03	0.02	-
rs1596274	2:185283979	T/C	0.9 (0.85-0.96)	1.43E-03	0.15	rs35943449
rs78724593	10:123561253	G/C	0.79 (0.68-0.91)	1.44E-03	0.02	-
rs62364586	5:93720171	T/C	1.3 (1.1-1.52)	1.68E-03	0.02	-
rs71407346	15:91454837	C/T	1.14 (1.05-1.24)	1.69E-03	0.08	rs71407347
rs41457849	1:61831219	T/C	1.17 (1.06-1.29)	1.82E-03	0.06	-
rs117255495	14:55268294	T/C	0.84 (0.75-0.94)	1.83E-03	0.04	-
rs2071240	5:149755744	G/C	0.87 (0.8-0.95)	1.91E-03	0.07	rs148432105; rs78451313; rs75194147; rs74555574; rs7728652; rs79012265; rs2295223; rs115781303; rs80008426; rs79083653; rs79972705
rs17064844	8:2123865	G/C	0.9 (0.84-0.96)	1.97E-03	0.13	-
rs187763209	11:33108014	G/A	0.75 (0.62-0.9)	2.09E-03	0.01	rs139071483; rs190807245; rs140779429; rs188476796; rs145819397; rs138727904; rs138163012; rs145706990; rs150487468; rs188505360; rs150201139; rs182531058; rs144216729; rs189433203; rs142250164; rs180831300; rs147374678; rs183359932; rs143626152; rs186219082

Continues in the next page

aSNP	position	M/m	OR (95%C.I.)	P	MAF	aSNPs in LD with SNP in first column
rs79420240	1:216112462	C/G	0.81 (0.71-0.93)	2.16E-03	0.03	rs79835717
rs114957082	6:1338570	G/A	1.11 (1.04-1.18)	2.62E-03	0.14	rs77510766; rs79616889; rs6934726; rs6939790; rs74447631; rs17261011; rs77926136; rs80181719; rs74484590; rs75289435; rs79565252; rs17260983; rs79842006; rs74622239; rs79368490; rs77796590; rs75802256; rs4959546; rs75586483; rs79783857; rs78451852; rs75472582; rs78252540; rs79618067; rs17261109; rs17261060; rs17201698
rs2088895	5:4728211	C/G	0.86 (0.78-0.95)	2.69E-03	0.05	-
rs112667742	2:157111093	G/C	1.15 (1.05-1.26)	2.74E-03	0.06	rs72892105; rs72892108; rs4369815; rs55723553; rs112576099; rs56273981; rs72892147; rs111506532; rs72902194; rs17248011; rs56004078; rs17188189; rs72904295; rs17188483; rs72909904; rs72909905; rs13410664; rs72906131; rs72909979; rs72906107; rs72906116; rs72907892; rs17188434; rs112939153; rs72906111; rs72902199
rs72729713	5:8063953	C/A	0.84 (0.74-0.94)	2.76E-03	0.04	-
rs4285939	12:119560691	G/T	0.82 (0.71-0.93)	3.18E-03	0.03	-
rs67388748	3:126954373	G/A	1.12 (1.04-1.21)	4.12E-03	0.10	rs68061258
rs11060965	12:131002001	C/T	1.13 (1.04-1.23)	4.16E-03	0.08	-
rs75804723	5:55983757	C/T	0.86 (0.77-0.95)	4.79E-03	0.05	rs75386672
rs72939407	18:60739269	C/G	1.14 (1.04-1.25)	4.93E-03	0.07	rs72939409; rs72935517
rs9893191	17:6346738	A/G	0.9 (0.83-0.97)	5.21E-03	0.09	rs4796495
rs138899456	12:63294090	C/T	0.77 (0.63-0.93)	5.90E-03	0.01	-
rs2700907	7:36189847	T/C	0.93 (0.89-0.98)	7.88E-03	0.26	-
